# Using Drop Jumps and Jump Squats to Assess Eccentric and Concentric Force-Velocity Characteristics

**DOI:** 10.3390/sports6040125

**Published:** 2018-10-24

**Authors:** Gavin L. Moir, Brandon W. Snyder, Chris Connaboy, Hugh S. Lamont, Shala E. Davis

**Affiliations:** 1 Department of Exercise Science, East Stroudsburg University of Pennsylvania, East Stroudsburg, PA 18301, USA; bsnyder16@po-box.esu.edu (B.W.S.); sdavis@esu.edu (S.E.D.); 2 Neuromuscular Research Laboratory, University of Pittsburgh, Pittsburgh, PA 15203, USA; connaboy@pitt.edu; 3 Department of Kinesiology, Coastal Carolina University, Conway, SC 29528, USA; hlamont@coastal.edu

**Keywords:** ground reaction force, power, absorption phase, propulsion phase

## Abstract

The purpose of this study was to investigate the eccentric and concentric force-velocity (Fv) characteristics recorded during drop jumps (DJ) from different heights and loaded jump squats (JS) and to determine the number of jumps required to accurately model the eccentric and concentric Fv relationships. Fourteen resistance-trained men (age: 21.9 ± 1.8 years) performed a countermovement jump (CMJ) and DJ from heights of 0.40, 0.60, and 0.80 m. JS with loads equivalent to 0%, 27%, 56%, and 85% 1-repetition maximum were performed in a separate session. Force platforms and a 3-D motion analysis system were used to record the average force ($\bar{F}$) and velocity ($\bar{v}$) during the absorption (CMJ, DJ_40_, DJ_60_, DJ_80_) and propulsion (JS_0_, JS_27_, JS_56_, JS_85_) phases of the jumps. Eccentric (absorption phase) and concentric (propulsion phase) Fv characteristics were then calculated and linear regression equations were determined when the number of jumps included was varied. $\bar{F}$ during the absorption phase significantly increased from CMJ to DJ_60_ while $\bar{v}$ increased significantly from CMJ to DJ_80_. The two-point method (CMJ, DJ_80_) resulted in a significantly lower *y*-intercept (mean difference [MD]: 0.7 N/kg) and a greater slope (MD: 0.7 Ns/m) for the eccentric Fv characteristics compared to the multiple-point method. $\bar{F}$ increased significantly and $\bar{v}$ decreased significantly with increasing external load in the JS conditions. The two-point method (JS_0_, JS_85_) resulted in a significantly greater *y*-intercept (MD: 1.1 N/kg) compared to the multiple-point method for the concentric Fv characteristics. Both DJ and loaded JS may provide means of assessing the eccentric and concentric Fv characteristics with only two jumps being required.

## 1. Introduction

Power output is an important determinant in many sports [1] and is dependent upon the specific force-velocity (Fv) characteristics of each athlete [2]. Concentric Fv characteristics can be described by an inverse linear relationship derived from measurements taken during the propulsion (concentric) phase of a series of loaded jump squats [3,4,5]. This relationship is predicated on the increased external load resulting in greater ground reaction forces (GRF) being produced but with lower vertical velocities attained by the load, thereby reflecting the inverse Fv relationship observed during concentric contractions of isolated skeletal muscle [6]. Furthermore, the linearity of the concentric Fv relationship assessed from loaded vertical jumps has led to the proposal that the use of only two distinct loads is required for the measurement of concentric Fv characteristics [3]. This two-point method of assessment is becoming a common means by which to measure concentric Fv characteristics and has been shown to be reliable, although it is recommended that the two loads selected must produce very different average velocities (e.g., using loads equivalent to 20% and 70% 1-repetition maximum [1-RM]; using loads that produce average velocities of ~1 m/s and ~0.5 m/s) in order to accurately and reliably model the Fv relationship [7,8]. Such methods have been previously validated in vertical jump tasks [9] and the resulting concentric Fv relationship has been used effectively to establish individualized resistance training programs to mitigate either force or velocity deficits [10].

Recently, researchers have promoted the importance of the eccentric capabilities of athletes in sports performance [11,12]. Given this proposed importance, the assessment of the eccentric Fv characteristics of athletes would appear pertinent. Eccentric capabilities are typically assessed using isokinetic dynamometry, whereby the velocity of lengthening is controlled during the task and force increases with velocity [13,14]. However, these assessments are limited to single joint tasks. Through the use of force platforms and motion analysis technologies it may be possible to assess the eccentric Fv characteristics through the use of drop jumps (DJ) completed from different drop heights. During the ground contact phase of a DJ task, the performer will proceed from an absorption to a propulsion phase as part of the stretch shortening cycle, with the absorption phase beginning when the athlete first contacts the ground and ending when the vertical motion of the center of mass has been arrested [1]. The absorption phase during a DJ task represents a time when the active musculotendinous units are lengthening (absorbing energy) and could perhaps reveal important information about the eccentric capabilities of the performer. For example, it has been shown that the vertical GRF during ground contact increases with increasing drop height during drop landing and DJ tasks [15,16,17,18,19]. The velocity of the center of mass (CM) attained during the absorption phase of ground contact would also increase with drop height, given the dependence of the kinetic energy of the CM at the beginning of ground contact upon the height of the drop [1], and this has been revealed in the extant literature [16,18]. This concomitant increase in both the force and velocity during the absorption phase of DJ tasks performed from increasing drop heights would theoretically mimic the increasing force and velocity observed during the eccentric portion of the force-velocity relationship of isolated skeletal muscle [6]. To date, however, the Fv characteristics during the absorption phase of DJ tasks have not been investigated. Therefore, the purpose of this study was to investigate the eccentric and concentric Fv characteristics recorded during drop jumps (DJ) from different heights and loaded jump squats (JS) and to determine the number of jumps required to accurately model the eccentric and concentric Fv relationships.

## 2. Materials and Methods

### 2.1. Subjects

Fourteen resistance-trained men (age: 21.9 ± 1.8 years; height: 1.79 ± 0.07 m; mass: 83.8 ± 8.6 kg; 1-repetition maximum [1-RM] parallel back squat: 162.0 ± 26.6 kg) volunteered to participate in this study, which was approved by the Institutional Research Board of East Stroudsburg University. All subjects reported having at least two years of exposure to regular resistance training (defined as a minimum of three resistance training sessions per week) and all were free from musculoskeletal injuries in the six month period prior to data collection.

### 2.2. Procedures

A counterbalanced cross-over design was employed with each subject attending two testing sessions. In one session each subject performed two trials of a CMJ and DJ from heights of 0.40 m (DJ_40_), 0.60 m (DJ_60_), and 0.80 m (DJ_80_), while in the other session each subject completed two JS with loads equivalent to 0% (JS_0_), 27% (JS_27_), 56% (JS_56_), and 85% (JS_85_) 1-RM. All jumps involved a countermovement, although the depth of the countermovement was not controlled during each jumping task. Furthermore, an arm swing was restricted during all jumps by having the subjects place their hands on their hips (CMJ and DJ) or by having the subjects hold a 1.70 m wooden dowel with a mass of 0.40 kg (JS_0_) or loaded Olympic barbell (loaded JS conditions) across the shoulders. Each subject completed two jumps under each condition, and the mechanical data were averaged across the two jumps. Prior to the jumps, each subject participated in a warm-up comprising dynamic activities (e.g., bodyweight squats, lunges, high kicks, high knees, repeated jumps in-place) and the subjects were required to perform a practice of each jump type prior to data collection. The instruction of “jump as high and as fast as you can” was also provided prior to the execution of each jump. During the DJ tasks, the subjects were instructed to step out from the box one foot at a time rather than jumping from the box.

### 2.3. Collection of Mechanical Data

Two force platforms (Kistler Type 9286AA; 1000 Hz) were used to record the vertical GRF during each jump from which the descent and ascent of the CM during ground contact was determined. The displacement of the CM during ground contact for all jumps was calculated through double integration of the net vertical GRF using the trapezoid method on the unfiltered digital signal. The absorption phase during the DJ tasks was determined when the net impulse of the vertical GRF (calculated as the time integral of the net vertical GRF using the trapezoid method) was positive and the CM was descending, while the propulsion phase during the JS tasks was determined when the net impulse of the vertical GRF was positive and the CM was ascending. These methods of determining the absorption and propulsion phases during ground contact were used to ensure that only force and velocity data were included in the calculations during the times when the subjects were actively generating force. The average vertical GRF during the absorption phase of CMJ and DJ tasks and during the propulsion phase of the JS tasks was normalized to body mass. The intraclass correlation coefficients (ICCs) for the normalized vertical force during the absorption phase of the tasks ranged from 0.92 to 0.99 (95% confidence limits [CL]: 0.77–1.00), while the ICCs for the normalized average vertical force during the propulsive phase ranged from 0.97 to 0.99 (95% CL: 0.92–1.00). The vertical velocity of the CM during ground contact was calculated as the time integral of the vertical GRF and was then averaged during the absorption (CMJ and DJ) and propulsion (JS) phases. The ICCs for the average vertical velocity during the absorption phase ranged from 0.84 to 0.94 (95% CL: 0.59–0.98) and those during the propulsion phase ranged from 0.70 to 0.96 (95% CL: 0.19–0.99).

The vertical velocity of the CM at initial ground contact during the DJ tasks was determined from a 3-D motion analysis system (Vicon, Oxford, UK; 200 Hz) that was synchronized with the force platforms. Specifically, the average vertical position of four retro-reflective markers placed around the pelvis of each subject (left and right anterior and posterior superior iliac spines) was differentiated with respect to time using the first central difference method to provide the vertical velocity of the CM as the subject descended from the plyometric boxes. It was assumed that the average location of the markers would reflect that of the CM, given that the jumps were performed with the hands placed on the hips. The raw position data was smoothed prior to differentiation using a generalized cross-validated quantic spline procedure. The vertical velocity at initial ground contact provided the constant of integration. Power output during the ground contact phase of all jumps was calculated as the product of the instantaneous vertical GRF and vertical velocity. The instantaneous power output was then averaged across the absorption and propulsion phases of the jumps and normalized to body mass. The ICCs for the normalized average power output during the absorption phases of the tasks ranged from 0.89 to 0.98 (95% CL: 0.69–0.99) while those for the normalized average power during the propulsion phase ranged from 0.86 to 0.98 (95% CL: 0.49–1.00).

### 2.4. Statistical Analysis

All statistical analyses were performed using the Statistical Package for the Social Sciences (SPSS version 20.0, Chicago, IL, USA). Measures of central tendency and spread of data were represented as means and standard deviations (±SD). A one-way ANOVA model with repeated measures on one factor (jump condition [4 levels]) was used to establish the differences in the mechanical variables (average force, average velocity, average power output) during the CMJ and DJs completed for the eccentric Fv characteristics, with a separate ANOVA performed for the concentric Fv characteristics derived from the JS tasks. Pairwise comparisons with Bonferroni corrections were used to establish where any significant differences were located. Linear regression equations were fitted to the Fv data for each subject using a different number of jumps for eccentric Fv characteristics (multiple-point method: CMJ, DJ_40_, DJ_60_, DJ_80_; two-point method: CMJ, DJ_80_) and the concentric Fv characteristics (multiple-point method: JS_0_, JS_27_, JS_56_, JS_85_; two-point method: JS_0_, JS_85_). Statistical differences in the parameters of *y*-intercept and slope between the two models for the eccentric and concentric Fv characteristics were determined using paired *t*-test. The magnitude of the relationship between the parameters calculated from each of the two models (multiple-point method vs. two-point method) was assessed using Pearson product moment correlation coefficients. The magnitudes of the coefficients were interpreted qualitatively using the categories recommended by Hopkins [20]: ≤0.1 was trivial; >0.1–≤0.3 was small; >0.3–≤0.5 was moderate; >0.5–≤0.7 was large; >0.7–≤0.9 was very large; >0.9–<1 was nearly perfect; 1 was perfect. The level of statistical significance for all analyses was set at *p* ≤ 0.05.

## 3. Results

The average force, velocity, and power output during the absorption phase of the CMJ and DJ tasks and the propulsion phase of the JS tasks are shown in Table 1 and Table 2, respectively.

The average force during the absorption phase of CMJ was significantly lower than the force during all DJ conditions (mean differences: 4.7–7.5 N/kg, *p* < 0.001), while that during DJ_40_ was significantly lower than that during both DJ_60_ and DJ_80_ (mean differences: 1.8 to 2.8 N/kg, *p* < 0.001). The average force during the absorption phase of DJ_60_ was not significantly different from that during DJ_80_ (mean difference: 0.9 N/kg, *p* = 0.220). The average vertical velocity (mean differences: 0.11 to 0.94 m/s, *p* < 0.05) and the average power output (mean differences: 5.5 to 32.2 W/kg, *p* < 0.001) increased significantly from CMJ to DJ_80_.

The average force during the propulsion phase of the JSs increased significantly as the external load increased (mean differences: 4.7 to 13.0 N/kg, *p* < 0.001) while the average vertical velocity (mean differences: −0.23 to −0.89 m/s, *p* < 0.001) and average power output (mean differences: −3.9 to −9.6 W/kg, *p* < 0.05) decreased significantly with the increasing load.

**Figure 1:**
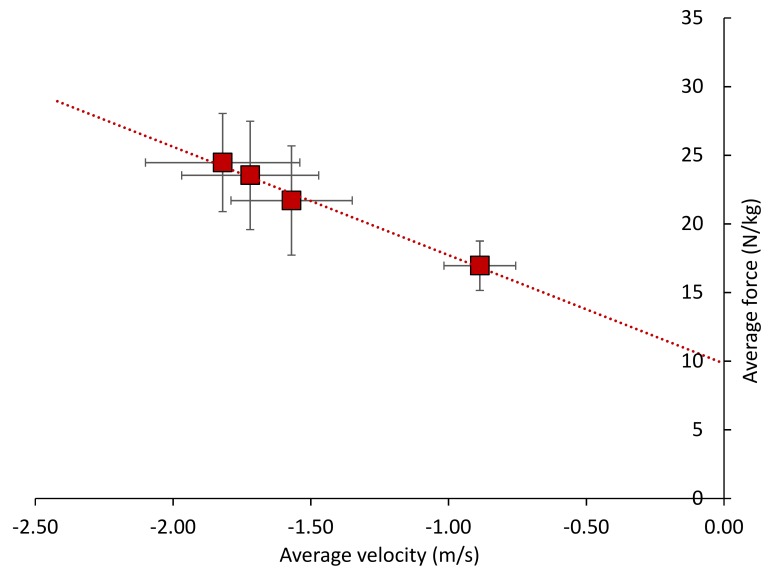
The eccentric force-velocity characteristics attained from a countermovement jump and three drop jumps from different heights. The solid squares are mean values; error bars are standard deviations. The dashed line represents the line of best fit.

Figure 1 shows the eccentric Fv relationship with all four jumps included (CMJ, DJ_40_, DJ_60_, DJ_80_) while the parameters for the two models of the eccentric Fv characteristics are shown in Table 3.

The *y*-intercept for the multiple-point method (CMJ, DJ_40_, DJ_60_, DJ_80_) was significantly greater than that for the two-point method (mean difference: 0.7 N/kg, *p* = 0.033), while the slope for the two-point method (CMJ, DJ_80_) was significantly greater than that for the multiple-point method (mean difference: 0.7 Ns/m, *p* = 0.029). The correlations for the *y*-intercept and slope derived from each of the two models were nearly perfect (0.93 and 0.96, respectively).

**Figure 2:**
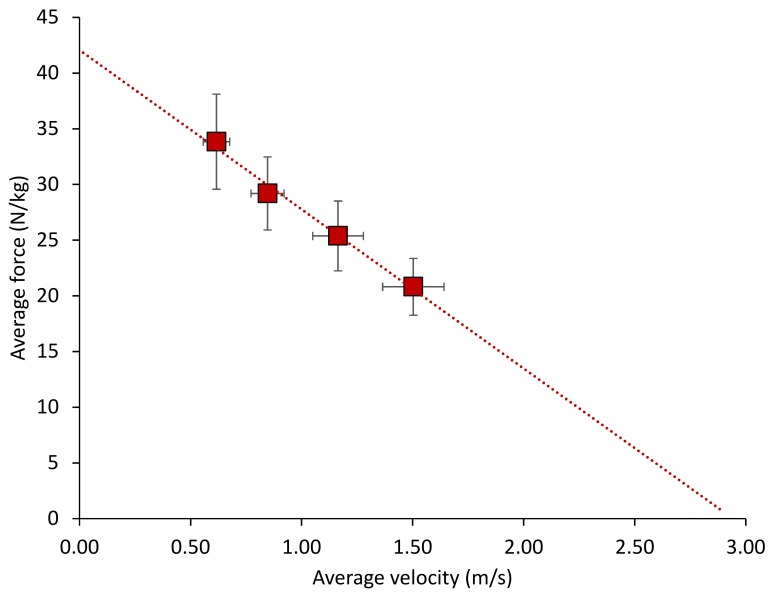
The concentric force-velocity characteristics attained from a series of unloaded and loaded jump squats. The solid squares are mean values; error bars are standard deviations. The dashed line represents the line of best fit.

Figure 2 shows the concentric Fv relationship with all four JS tasks included, while the parameters for the two models of the concentric Fv characteristics are shown in Table 4.

The *y*-intercept for the two-point method (JS_0_, JS_85_) was significantly greater than that for multiple-point method (mean difference: 1.1 N/kg, *p* = 0.009), but the correlation between the two was nearly perfect (0.98). The slope for the two-point method was greater than that for the multiple-point method, although the difference was not statistically significant (mean difference: 0.6 Ns/m, *p* = 0.051) and the correlation was nearly perfect (0.96).

## 4. Discussion

The purpose of this study was to investigate the eccentric and concentric Fv characteristics recorded during DJ from different heights and loaded JS and to determine the number of jumps required to accurately model the eccentric and concentric Fv relationships. The analysis of the two linear regression models for the eccentric Fv characteristics used in the present study revealed a significantly lower *y*-intercept and a significantly greater slope for the two-point method (CMJ, DJ_80_) compared to the multiple-point method. The *y*-intercept of the eccentric Fv relationship assessed using DJ tasks should be 9.81 N/kg, given that the average vertical force would be equal to bodyweight under conditions of null velocity and this intercept value would not be expected to change in response to physiological adaptations accrued from different training regimens. Although both models returned *y*-intercept values very close to 9.81 N/kg, the two-point method produced the closest value largely as a result of the inclusion of both the DJ_60_ and DJ_80_ tasks in the multiple-point method (these two jump conditions produced very similar average vertical forces despite significantly different average vertical velocities). The slope of the linear regression models reflect the balance between the force and velocity characteristics to the power output of the athlete being tested. This regression term would be expected to change in response to physiological adaptations that alter the eccentric force capabilities, as will be discussed below. However, the slope of the eccentric Fv relationship as measured through a series of DJ tasks would also be sensitive to any alterations in technique, including factors that would affect fall time (stepping down as opposed to stepping out from the box, excessive hip and knee flexion at ground contact). This highlights the requirement for the practitioner to provide appropriate instructions prior to the execution of the jumps (i.e., “step out from the box”, “jump as high and as fast as possible”) and to evaluate the technique accordingly so as to ensure that any alterations in the Fv characteristics are due to physiological adaptations rather than technical modifications. Related to this point, previous researchers have proposed that the instructions presented to the athlete are likely to be superseded by the constraints imposed by the DJ task, with greater drop heights necessarily inducing alterations in technique that would be associated with lower eccentric loading irrespective of the instructions presented to the athlete [21]. It may therefore be pertinent to limit the drop heights used in the DJ tasks in order to maintain the linearity of the Fv relationship. Furthermore, the constraints-led approach to movement control and coordination [1] would propose that physiological adaptations accrued from specific training regimens, such as increased musculotendinous stiffness, would be expected to influence the technical aspects of DJ performance. Future researchers should therefore assess the reliability of the eccentric Fv characteristics measured through a series of DJ tasks. It has recently been shown that the use of the two most distant loads may not be appropriate when using the two-point method to determine the Fv characteristics during a cycling task, as very high loads typically have low reliability [22]. Low reliability in the average force and velocity variables may be expected at greater drop heights, further limiting the drop heights selected when assessing eccentric Fv characteristics.

It is difficult to interpret the meaningfulness of the differences in the eccentric Fv characteristics between the two models presented in this study. The difference in the *y*-intercept (0.7 N/kg) is slightly greater than the smallest worthwhile change (SWC) of 0.5 N/kg (calculated as 20% of the between-subject standard deviation for the multiple-point method [23]), while the difference in the slope of 0.7 Ns/m is equal to the SWC. Clearly there is a need to assess the reliability of the eccentric Fv characteristics to establish the meaningfulness of the differences in the parameters when the relationship is modeled using a different number of jumps performed from different drop heights. Furthermore, the validity of the eccentric Fv requires investigation through comparison of athletes from different training backgrounds (discriminative validity) as well as the responses to different physiological adaptations induced through different training methods, particularly those that would influence vertical stiffness and therefore the average force generated during the absorption phase of the DJ tasks.

When assessing the concentric Fv through a series of loaded JS, in the present study it was found that the average vertical force during the propulsion phase increased significantly with the external load lifted, while both the average vertical velocity and average power output were decreased significantly. Such alterations in these mechanical variables have been reported previously [2,4,5,24] and form the basis of the inverse linear relationship between force and velocity measured through the use of loaded JS. Indeed, the linearity of the relationship has led to the proposal that the use of only two distinct loads is required for the measurement of concentric Fv characteristics [3], although the two loads chosen should be sufficiently different that they result in distinct vertical velocities [7,8]. It was found that the two-point method using two jumps that produced significantly different average vertical velocities (JS_0_, JS_85_) resulted in a significantly greater *y*-intercept than the multiple-point method used in the present study. The difference of 1.1 N/kg between the two models falls slightly below the SWC for the *y*-intercept of 1.2 N/kg and is approximately 3% of the group mean for the multiple-point method. Feeney et al. [4] reported a coefficient of variation (CV) of 10% for the *y*-intercept determined across consecutive trials of vertical jumps using nine different external loads (0–40% body mass). Jiménez-Reyes et al. [10] reported changes in the *y*-intercept ranging from 2% to 24% for groups undertaking different training regimens over a 9 week period, while Cuk et al. [2] reported that the *y*-intercept of strength-trained athletes was 37% greater than that of recreationally active individuals. Taken together, it can be concluded that the statistically significant difference between the *y*-intercept values of the two models assessed in the present study does not appear to be practically meaningful. The mean difference in the slope between the two models (0.6 Ns/m) is similarly unlikely to be meaningful, as it falls within the SWC of 0.6 Ns/m and is substantially less than the CV of 30% for the slope of the concentric Fv relationship determined across consecutive trials of loaded vertical jumps reported by Feeney et al. [4]. Furthermore, the difference reported in the present study is lower than the differences in the slope of the concentric Fv relationship observed following adaptations accrued from different training regimens and the difference in the slope between strength-trained and recreationally active individuals [2,10]. Therefore, the use of the two-point method to determine the concentric Fv characteristics from a series of unloaded and loaded JS would appear to be appropriate, as proposed by previous researchers [3,9].

It was found in the present study that the average vertical ground reaction force during the absorption phase increased significantly as the drop height increased from the CMJ to DJ_60_, while the increase in the average force between DJ_60_ and DJ_80_ was not statistically significant. The work-energy theorem holds that the work done during the absorption phase of ground contact is equivalent to the kinetic energy of the CM at initial ground contact and that the kinetic energy at ground contact is equal to the gravitational potential energy of the CM when the athlete is stood atop the box (such relationships can be derived from equations of motion [1]). Given that the work done will be equal to the product of the average force acting on the CM during the absorption phase and the displacement of the CM [1], an increase in the average force exerted by the performer during absorption would be expected if the performer were to maintain vertical stiffness across the increasing drop heights. Previous researchers have indeed noted that force during ground contact increases with increasing drop height during drop landing and DJ tasks [15,16,17,18,19]. It has also been shown that the drop height associated with DJ tasks influences the neuromuscular strategies used during ground contact that affect the stiffness of the musculotendinous units [25,26,27]. Specifically, DJ tasks performed from lower drop heights (e.g., 0.30 m) have been proposed to emphasize the short latency response of the active musculature and the excitability of the H-reflex, while DJ tasks performed from greater drop heights (e.g., 0.80 m) result in reduced muscular activity and lower excitability and therefore lower stiffness of the musculotendinous units. It is also important to note that the average force exerted during the absorption phase of DJ tasks is also likely to be sensitive to changes in technique. For example, alterations in the vertical GRF have been shown to accompany changes in the position of the foot at initial contact with the ground during DJ tasks [28]. These changes in force were associated with changes in vertical stiffness. Moreover, decreases in the magnitude of the GRF during landing tasks have been reported following the presentation of the instruction to “land softly”, causing performers to reduce their stiffness upon landing [29,30]. Therefore, the lack of significant increase in the average vertical force between DJ_60_ and DJ_80_ in the present study may reflect either a limit to the eccentric capabilities of the subjects (reduced spinal excitability) or a conscious strategy of altering landing posture to reduce stiffness, and therefore the force during the DJ_80_ task, thereby limiting the forces experienced during impact.

The average vertical velocity of the CM during the absorption phase of the DJ tasks increased significantly with drop height in the present study. Evidence for the dependence of the vertical velocity during the absorption phase of DJ tasks on drop height has been provided by the data of Bobbert et al. [16] and Peng [18] who reported an increase in the normalized impulse during the absorption phase as the drop height increased from 0.20 m to 0.60 m. The dependence of average velocity on drop height follows from the work-energy theorem, which states that the kinetic energy of the CM at ground contact is equal to the gravitational potential energy of the CM when the athlete is atop the box and that the work performed on the CM during the absorption phase will reduce the kinetic energy to zero [1]. Therefore, the average velocity during the absorption phase of DJ tasks will be largely unaffected by biomechanical factors including the stiffness of the performer during ground contact, being influenced only by the height of the drop. Indeed, in the present study it was found that the power output during the DJ_80_ was significantly higher than that during DJ_60_ largely due to the greater average vertical velocity attained during the absorption phase resulting from the greater drop height. (It should be noted that there are other factors beyond the height of the drop that could influence the average velocity during absorption, such as the technique used when stepping from the box and the posture adopted at initial ground contact, both of which would affect the duration of the fall and therefore the kinetic energy of the CM at ground contact). It is therefore likely that the eccentric Fv characteristics recorded during a series of DJ tasks from different drop heights will be most reflective of an athlete’s force characteristics and might be sensitive to adaptations in biomechanical variables such as vertical stiffness accrued from training regimens including plyometric exercises [31] and eccentric resistance exercises [32]. In contrast, the average velocity during the absorption phase of DJ tasks would not be expected to change in response to physiological adaptations if the drop heights used and the athletes’ technique (e.g., stepping from the box, posture at landing) remained consistent across the testing period. It is interesting to note that the isokinetic methods that have typically been employed to assess the eccentric Fv characteristics rely upon a series of fixed lengthening velocities with eccentric force expected to change under each velocity condition [13,14]. The present methods utilizing DJ from different heights follow this principle.

## 5. Conclusions

The use of CMJ and DJ from different drop heights and a series of unloaded and loaded JS provide a means of assessing the eccentric and concentric Fv characteristics, with the requirement for only two jumps to be performed. However, practitioners need to ensure that the technique used when executing the jumps remains consistent.

## Figures and Tables

**Table 1:** The average force, average velocity, and average power output during the absorption phase of the countermovement jump and the drop jumps from different heights. Values are means ± standard deviations.

Mechanical Variable	Jump Condition			
	CMJ	DJ_40_	DJ_60_	DJ_80_
Average force (N/kg)	17.0 ± 1.8	21.7 ± 4.0	23.5 ± 4.0	24.5 ± 3.6
Average velocity (m/s)	−0.89 ± 0.13	−1.57 ± 0.22	−1.72 ± 0.25	−1.82 ± 0.28
Average power (W/kg)	−13.7 ± 3.0	−32.9 ± 5.5	−40.3 ± 6.4	−45.8 ± 7.6

CMJ is countermovement jump; DJ_40_ is drop jump from a 0.40 m drop height; DJ_60_ is drop jump from a 0.60 m drop height; DJ_80_ is drop jump from a 0.80 m drop height.

**Table 2:** The average force, average velocity, and average power output during the propulsion phase of the unloaded and loaded jump squats. Values are means ± standard deviations.

Mechanical Variable	Jump Condition			
	JS_0_	JS_27_	JS_56_	JS_85_
Average force (N/kg)	20.8 ± 2.6	25.4 ± 3.1	29.2 ± 3.3	33.8 ± 4.3
Average velocity (m/s)	1.50 ± 0.14	1.16 ± 0.11	0.85 ± 0.07	0.62 ± 0.06
Average power (W/kg)	30.9 ± 5.7	29.7 ± 5.6	25.2 ± 4.6	21.3 ± 3.6

JS_0_ is unloaded jump squat; JS_27_ is jump squat with a load equivalent to 27% 1-repetition maximum (1-RM); JS_56_ is jump squat with a load equivalent to 56% 1-RM; JS_85_ is jump squat with a load equivalent to 85% 1-RM.

**Table 3:** The model parameters and 95% confidence limits for the eccentric force-velocity characteristics recorded from a countermovement jump and drop jumps from different heights. Values are means ± standard deviations.

Model	Jumps Included	*y*-Intercept (N/kg)	95% CL	Slope (Ns/m)	95% CL
Multiple-point	CMJ, DJ_40_, DJ_60_, DJ_80_	10.3 ± 2.7	7.5–16.6	−8.0 ± 3.5	−5.8 to −12.9
Two-point	CMJ, DJ_80_	9.6 ± 3.0	7.0–15.5	−8.7 ± 3.5	−6.3 to −14.0

CMJ is unloaded jump squat; DJ_40_ is drop jump from a 0.40 m drop height; DJ_60_ is drop jump from a 0.60 m drop height; DJ_80_ is drop jump from a 0.80 m drop height; 95% CL is 95% confidence limits.

**Table 4:** The model parameters and 95% confidence limits for the concentric force-velocity characteristics recorded from a series of unloaded and loaded jump squats. Values are means ± standard deviations.

Model	Jumps Included	*y*-Intercept (N/kg)	95% CL	Slope (Ns/m)	95% CL
Multiple-point	JS_0_, JS_27_, JS_56_, JS_85_	41.7 ± 6.1	30.2–67.2	−14.2 ± 3.2	−10.3 to −22.9
Two-point	JS_0_, JS_85_	42.9 ± 6.5	31.1–69.1	−14.8 ± 3.8	−10.7 to −23.8

JS_0_ is unloaded jump squat; JS_27_ is jump squat with a load equivalent to 27% 1-repetition maximum; JS_56_ is jump squat with a load equivalent to 56% 1-repetition maximum; JS_85_ is jump squat with a load equivalent to 85% 1-repetition maximum; 95% CL is 95% confidence limits.
